# Real-world evidence on the strategy of olmesartan-based triple single-pill combination in Korean hypertensive patients: a prospective, multicenter, observational study (RESOLVE-PRO)

**DOI:** 10.1186/s40885-021-00177-z

**Published:** 2021-11-01

**Authors:** Il Suk Sohn, Sang-Hyun Ihm, Gee Hee Kim, Sang Min Park, Bum-Kee Hong, Chang Hoon Lee, Sang Hyun Lee, Dae-Il Chang, Sung-Pil Joo, Sang-Chan Lee, Yong-ho Lee, Dong Woon Jeon, Kyung Tae Jung, Si Jae Rhee, Yoon-Jin Cho, Chong-Jin Kim, Investigators, Yun Sung Kim, Seong-Ill Woo, Kyounghoon Lee, Jung Ho Heo, Sang-Ho Park, Euy Jin Choi, Sun Ho Hwang, Yonh-Gu Chung, Young Jin Youn, Eul-Soon Im, Jong Sam Baik, Nack-Cheon Choi, Jin Bae Lee, Kyu-Hyung Ryu, Ji-Yong Jang, Sung-Ji Park, Dong-Ick Shin, Jin Oh. Na, Yun Jae Chung, Su Hyun Kim, Jong-Young Lee, Yoon-Sang Oh, Jong Hwan Choi, Hye Sun Seo, Su Kyoung Kwon, Hyung-Jun Kim, Jin-Sun Park, Kyoung-Ha Park, Kyoung-Soo Lee, Sung Chang Chung, Han-Jin Cho, Chang-Min Lee, Sung-Rae Kim, Jinkwon Kim, Hyeong-gyun Oh, Ik Seong Park, Pil-Wook Chung, Sung-Rae Cho, Hyun-Seung Kang, Seung Jin Lee, Kyungil Park, Si Won Lee, Jang-Won Son, Soo Kyoung Kim, Joong-Hwa Chung, Jin-Soo Byon, Hong Euy Lim, Bum-Tae Kim, Yu Jeong Choi, Soe Hee Ann, Sang Min Sung, Jun-Hee Lee

**Affiliations:** 1 Department of Internal Medicine, Division of Cardiology, Kyung Hee University Hospital at Gangdong, Seoul, Republic of Korea; 2 Department of Internal Medicine, Division of Cardiology, Bucheon St. Mary’s Hospital, College of Medicine, The Catholic University of Korea, Bucheon, Republic of Korea; 3 Department of Internal Medicine, Division of Cardiology, St. Vincent’s Hospital, Suwon, Republic of Korea; 4 Department of Cardiology, Nowon Eulji Medical Center, Eulji University, Seoul, Republic of Korea; 5 Division of Cardiology, Heart Center, Gangnam Severance Hospital, Yonsei University College of Medicine, Seoul, Republic of Korea; 6 Department of Internal Medicine, Division of Cardiology, Veterans Health Service Medical Center, Seoul, Republic of Korea; 7 Department of Internal Medicine, Division of Cardiology, Pusan National University Yangsan Hospital, Yangsan, Republic of Korea; 8 Department of Neurology, Kyung Hee University Hospital, Seoul, Republic of Korea; 9 Department of Neurosurgery, Chonnam National University Hospital, Chonnam National University Medical School, Gwangju, Republic of Korea; 10 Department of Neurology, Dong-Eui Hospital, Busan, Republic of Korea; 11 Department of Internal Medicine, Yonsei University College of Medicine, Seoul, Republic of Korea; 12 Department of Internal Medicine, Division of Cardiology, National Health Insurance Service Ilsan Hospital, Goyang, Republic of Korea; 13 Department of Internal Medicine, Division of Cardiology, Eulji University Hospital, Daejeon, Republic of Korea; 14 Medical Affairs Department, Daiichi Sankyo Korea Co., Ltd., Seoul, Republic of Korea; 15 Department of Cardiology, CHA Gangnam Medical Center, CHA University School of Medicine, Seoul, South Korea

**Keywords:** Hypertension, Observational study, Olmesartan, Amlodipine, Hydrochlorothiazide, Real-world, Single-pill combination, Korea

## Abstract

**Background:**

In this prospective, multicenter, non-comparative observational study, the effectiveness and safety of the triple single-pill combination (SPC) of olmesartan/amlodipine/hydrochlorothiazide (OM/AML/HCTZ) were evaluated in a real clinical practice setting in Korean patients with essential hypertension.

**Methods:**

A total of 3752 patients were enrolled and followed for 12 months after administration of OM/AML/HCTZ. Primary endpoint was change from baseline to month 6 in the mean systolic blood pressure (SBP). Secondary endpoints included changes from baseline in the mean SBP at month 3, 9, 12 and the mean diastolic blood pressure (DBP) at month 3, 6, 9, 12; changes in the mean SBP/DBP according to age and underlying risk factors; and blood pressure control rate (%) at different time points. Adherence to and satisfaction with OM/AML/HCTZ treatment among patients and physicians were assessed by medication possession ratio (MPR) and numeric rating scale, respectively, as exploratory endpoints. Safety was evaluated by the incidence and severity of adverse events (AEs) as well as the discontinuation rate due to AEs.

**Results:**

OM/AML/HCTZ administration led to significant reductions in the mean SBP/DBP by 11.5/6.6, 12.3/7.0, 12.3/7.2, and 12.8/7.4 mmHg from baseline to month 3, 6, 9 and 12, respectively (*P* < 0.0001). The BP reductions were maintained throughout the 1-year observation period in all patients with different age groups and risk factors (diabetes mellitus, cardiovascular disease, and renal disease). The BP control rate (%) of < 140/90 mmHg was 65.9, 67.9, 68.9, and 70.6% at month 3, 6, 9, and 12, respectively. The mean MPR during the observation period was 0.96. The safety results were consistent with the previously reported safety profile of OM/AML/HCTZ.

**Conclusions:**

Treatment with the triple SPC of OM/AML/HCTZ demonstrated significant effectiveness in reducing SBP/DBP and achieving target BP control with high adherence over the 1-year observation period in Korean hypertensive patients and was well-tolerated.

**Trial registration:**

CRIS, KCT0002196, Registered 3 May 2016.

**Supplementary Information:**

The online version contains supplementary material available at 10.1186/s40885-021-00177-z.

## Background

Hypertension is a critical public health burden affecting approximately 1.4 billion individual worldwide and well-known significant risk factor for cardiovascular disease (CVD), such as myocardial infarction, stroke, and heart failure [1]. There is compelling evidence that treatment of hypertension reduces the risks of CVD, cerebrovascular disease, and all-cause mortality. However, despite the clear benefits of effective blood pressure (BP) control, the number of hypertensive patients in Korea has been increased to nearly 11 million, while the control rate of hypertension has remained suboptimal over a period of 10 years (44% in 2007 vs. 41% in 2016) [2].

Lack of patient adherence to antihypertensive therapy has been considered as a major impediment to effective hypertension management. Multiple strategies have been developed to improve patient adherence and hence BP control. It has been well-known that combining antihypertensive agents from different classes produces a synergistic effect in reducing BP, which is estimated approximately five times more effective than increasing the dose of a single agent [3, 4]. Reflecting this, recent hypertension guidelines have recommended the use of a single-pill combination (SPC) therapy to initiate antihypertensive therapy in most patients, with a preferred combination of Renin-Angiotensin System blocker and calcium channel blocker and/or thiazide/thiazide-like diuretic [5, 6]. The triple SPC of olmesartan, amlodipine and hydrochlorothiazide (OM/AML/HCTZ) consists of the combination recommended by current guidelines and has shown to improve patient medication adherence compared with an extemporaneous combination (55.1% vs. 15.9%, *P* < 0.0001, respectively) [7].

The efficacy and safety of the SPC of OM/AML/HCTZ have been previously determined in a number of randomized-controlled trials (RCTs), including 12-week TRINITY study, a global phase III clinical trial of OM/AML/HCTZ, and 40-week open label extension of TRINITY study [8, 9]. However, it is difficult to apply the results of the global studies to Korean patients in general as the proportion of Asian population included in those studies was small. Previous observational studies on OM/AML/HCTZ also lacked data on Asian ethnicity, particularly Koreans, and have been carried out over short periods [10, 11].

Real-world evidence (RWE) has been highlighted due to its ability to represent the actual clinical practice. Recently, RESOLVE study retrospectively investigated the effectiveness and safety of OM/AML/HCTZ in Korean hypertensive patients in a real clinical setting [12]. Since RWE of the use of triple SPC in Korean patients is still limited, this study prospectively evaluated the effectiveness and safety of OM/AML/HCTZ in Koreans patients with essential hypertension in a real-world practice in conjunction with the retrospective cohort study (RESOLVE-PRO).

## Methods

### Study population

Korean patients with essential hypertension who had initiated OM/AML/HCTZ at physician’s discretion according to routine clinical considerations were included in this study. Neither prior treatment nor SPC treatment were restricted. Patients who were treatment-naïve were also included. Patients or their legal guardians consented to study participation after being informed about the protocol, collection of patient data, the effect, and possible adverse reactions (ADRs) of OM/AML/HCTZ. Patients were excluded if they had received OM/AML/HCTZ within 3 months prior to enrollment, if they were participating in another clinical study, or were deemed ineligible for this study at the discretion of the treating physician.

### Study design and procedures

This study was a prospective, multicenter, non-comparative observational study to investigate the effectiveness and safety of OM/AML/HCTZ in a real clinical setting. Patient enrollment began on May 3, 2016 and the last follow-up was completed on January 28, 2019. A total of 70 investigators from 66 medical departments of 55 institutions participated in the study. BP was measured in a clinical setting of each institution in accordance with the standard measurement method of clinic BP from the 2018 Korea Society of Hypertension (KSH) guidelines [6]. This study was conducted in accordance with Declaration of Helsinki and the relevant pharmaceutical affairs law in Korea. Written approval of the study protocol and informed consent procedures were obtained from the Institutional Review Board (IRB) of all participating institutions.

Dose of OM/AML/HCTZ (20/5/12.5 mg, 40/5/12.5 mg and 40/10/12.5 mg are approved doses in Korea) and the frequency of follow-up were determined at the discretion of the treating physician. Apart from the routine 3-month follow-up, the study involved no additional visits, treatments or procedures required beyond those occurring within the course of normal care.

Collected data included patient demographic characteristics, hypertension-related information, underlying diseases, concomitant drug use, medical history, and prescription information. Data on additional antihypertensive medications or dose escalation of OM/AML/HCTZ in patients with inadequate BP control were also collected.

### Effectiveness evaluation

The effectiveness analysis set included patients who met enrollment criteria and received follow-up assessment at least once during the observation period after administration of OM/AML/HCTZ at baseline visit. The safety analysis set included patients who had administered OM/AML/HCTZ at least once during the observation period.

The primary endpoint was the change from baseline in the mean systolic BP (SBP) at month 6 following administration of OM/AML/HCTZ. The secondary endpoints included the changes in the mean SBP at month 3, 9, 12 and the mean diastolic BP (DBP) at month 3, 6, 9, 12 compared to baseline; the changes in the mean SBP/DBP according to age and accompanying risk factors; and the rate of BP control at each visit. The rate of BP control was defined as the proportion of patients who had achieved SBP/DBP below the target of < 140/90 mmHg during the observation period. The target BP goal of SBP/DBP < 140/90 mmHg was set according to 2018 KSH and 2018 European Society of Cardiology (ESC)/European Society of Hypertension (ESH) guidelines at the time of analysis [5, 6].

For exploratory endpoints, patient medication adherence was assessed using medication possession ratio (MPR). The MPR was calculated as a proportion, representing the number of days covered by prescription of OM/AML/HCTZ, divided by the number of days between the date of the first prescription and the date for the last prescription. In addition, patients’ and physicians’ satisfaction on the use of OM/AML/HCTZ at month 6 and 12 were measured using an eleven-point numeric rating scale (NRS) (ranging from 0 = ‘not satisfied at all’ to 10 = ‘completely satisfied’).

### Safety evaluation

Safety evaluation of OM/AML/HCTZ included the incidence and severity of adverse events (AEs) and ADRs, as well as the discontinuation rate of OM/AML/HCTZ due to AEs.

### Statistical analysis

Unless specified otherwise, two-sided tests were used for all statistical analyses using SAS ver. 9.4 (SAS Institute, Cary, NC, USA), with a level of significance level set at 5% (*P* < 0.05). Descriptive statistics including number of participants, mean and standard deviation were used for continuous data, while categorical data were presented using frequency and ratio (%). The distributions of all continuous variables were tested for normality; parametric tests were performed for variables with a normal distribution, whereas non-parametric tests were for those without. The normality of data distribution was examined using the Shapiro-Wilk test with a *P*-value less than 5% (*P* < 0.05). Paired t-tests were performed for variables with a normal distribution, while Wilcoxon signed-rank tests and McNemar’s tests were performed for those without. *P*-values were presented for these variables. No adjustments were made for missing data from continuous variables at particular time points; missing data due to patient dropout prior to completion of the study; and missing data from the safety analysis set.

## Results

### Study population

**Fig. 1:**
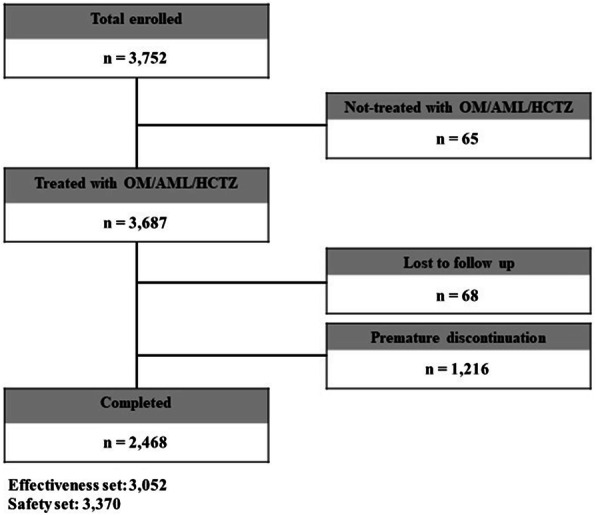
Study disposition. All patients with essential hypertension who had initialed olmesartan/amlodipine/hydrochlorothiazide (OM/AML/HCTZ) at physician’s discretion according to routine clinical considerations were included in this study. The effectiveness analysis set included patients who met enrollment criteria and received follow-up assessment at least once during the observation period after administration of OM/AML/HCTZ at baseline. The safety analysis set included patients who had administered OM/AML/HCTZ at least once during the observation period

A total of 3752 patients were enrolled from 55 institutions between May 2016 and January 2019. Of the patients enrolled, 3687 patients (98.3%) had administered OM/AML/HCTZ and 2468 patients (65.8%) completed the study. The effectiveness analysis set included 3052 patients (81.3%) and the safety analysis set included 3370 patients (89.8%) (Fig. 1).

The patient demographic characteristics are summarized in Table 1. The mean age of the enrolled patients was 62.7 ± 13.3 years and 49.2% were aged 65 years or older. A total of 57.2% of them were male and 41.4% were female. The mean height, body weight, and body mass index were 163.0 ± 9.3 cm, 70.1 ± 14.0 kg, and 26.1 ± 4.1 kg/m^2^, respectively. The mean duration of hypertension was 9.3 ± 8.1 years. The mean SBP and DBP at baseline were 143.6 ± 19.2 mmHg and 83.6 ± 14.0 mmHg, respectively. Of the enrolled patients, 17.7% (*n* = 663) were smokers, 28.1% (*n* = 1053) consumed alcohol, and 19.5% (*n* = 731) had a family history of hypertension. A total of 63.4% (*n* = 2378) of the enrolled patients had underlying risk factors including others (*n* = 1597), diabetes mellitus (DM; *n* = 1231), and renal disease (RD; *n* = 141) (Table 1).

**Table 1:** Demographic and baseline characteristics (enrolled set)

Characteristic	Enrolled set (*n* = 3752)
Age (yr)	62.7 ± 13.3
Age group (yr)	
< 60	1395 (37.2)
60–64	458 (12.2)
65–69	552 (14.7)
70–74	548 (14.6)
75–80	499 (13.3)
> 80	247 (6.6)
Elderly age (≥65 yr)	1846 (49.2)
Sex	
Male	2145 (57.2)
Female	1554 (41.4)
Baseline height (cm), *n* = 2917	163.0 ± 9.3
Baseline body weight (kg), *n* = 2931	70.1 ± 14.0
BMI (kg/m^2^), *n* = 2877	26.1 ± 4.1
Baseline SBP (mmHg), *n* = 3640	143.6 ± 19.2
Baseline DBP (mmHg), *n* = 3638	83.6 ± 14.0
Hypertension period (yr), *n* = 2603	9.3 ± 8.1
Smoking status, smoker	663 (17.7)
Alcohol consumption, yes	1053 (28.1)
Family history of hypertension, yes	731 (19.5)
Accompanying risk factor, yes	2378 (63.4)
Accompanying risk factor	
Renal disease	141 (3.8)
Diabetes	1231 (32.8)
Others	1597 (42.6)

Data are presented as mean ± standard deviation or number (%). Hypertension period (mo) = (enrollment date – date of hypertension diagnosis + 1) / 30.44. The date of hypertension diagnosis was assumed to be the first day of the month in the recorded year. Duplicates were allowed for accompanying risk factors

*BMI* body mass index, *SBP* systolic blood pressure, *DBP* diastolic blood pressure

### Effectiveness

**Fig. 2:**
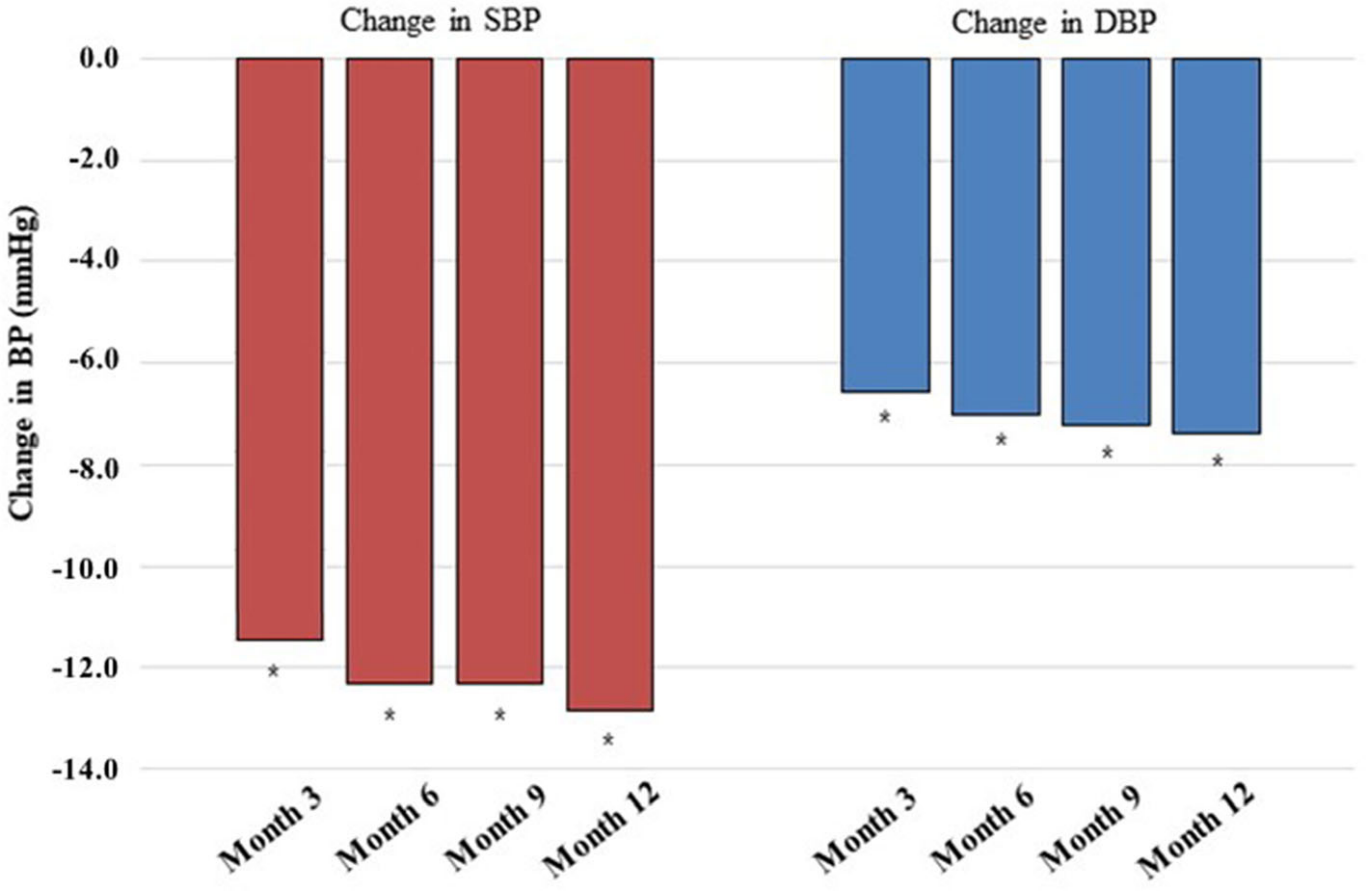
Changes from baseline in the mean SBP/DBP at month 3, 6, 9, and 12 after olmesartan/amlodipine/hydrochlorothiazide (OM/AML/HCTZ) administration. Effectiveness set (*n* = 3052). BP, blood pressure; SBP, systolic BP; DBP, diastolic BP. *P < 0.0001 (Wilcoxon singed-rank test)

**Fig. 3:**
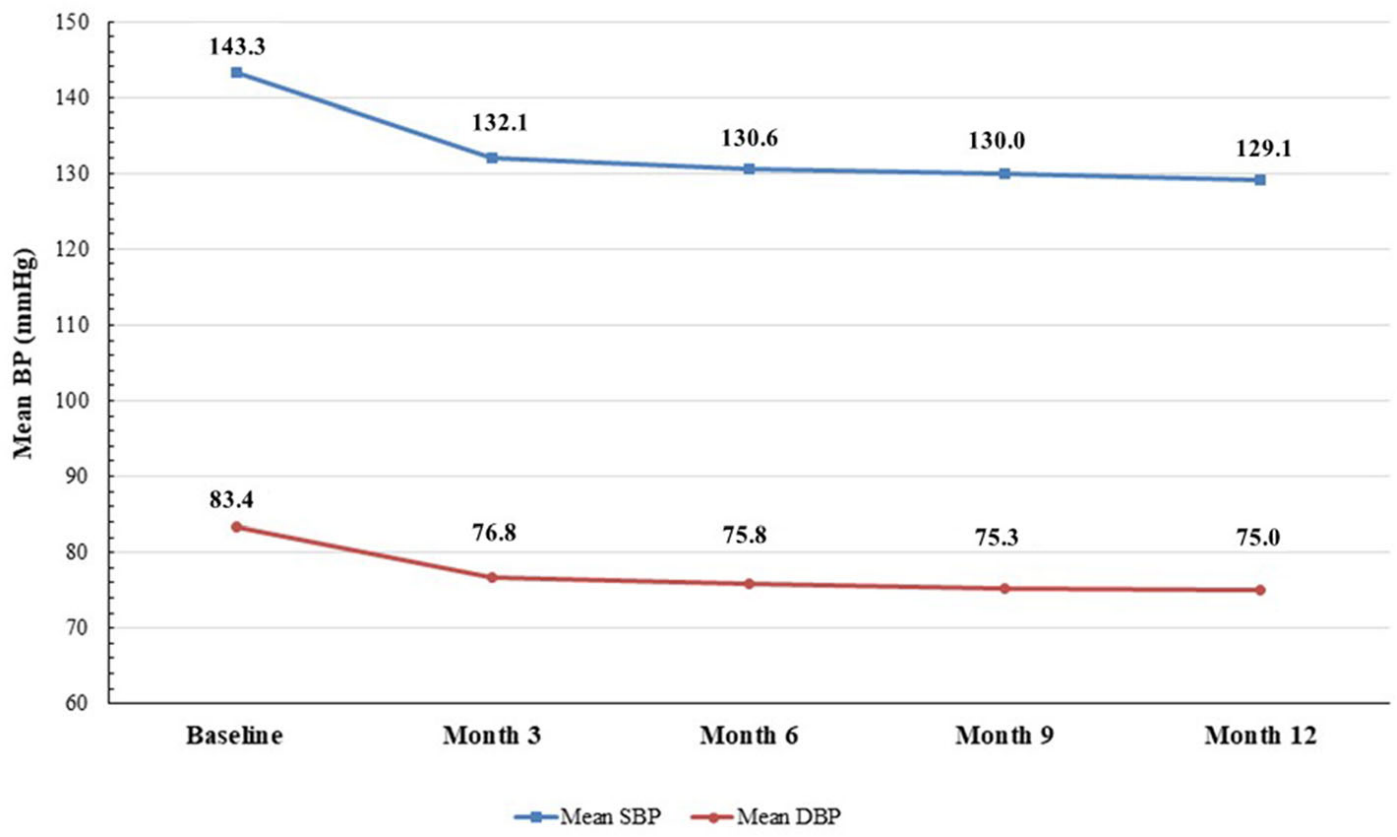
Time course change from baseline in the mean SBP/DBP at month 3, 6, 9, and 12 after olmesartan/amlodipine/hydrochlorothiazide (OM/AML/HCTZ) administration. Effectiveness set (*n* = 3052). BP, blood pressure; SBP, systolic BP; DBP, diastolic BP

In the effectiveness analysis set (*n* = 3052), the mean SBP was significantly reduced by 12.3 ± 20.8 mmHg from baseline (143.3 ± 19.4 mmHg) to 6 months (130.6 ± 16.1 mmHg) after administration of OM/AML/HCTZ (*P* < 0.0001). The reductions in the mean SBP at month 3, 9 and 12 were as follows: 11.5 ± 20.4 mmHg, 12.3 ± 20.7 mmHg, and 12.8 ± 20.4 mmHg, respectively (all P < 0.0001). The mean baseline DBP was also significantly reduced from baseline (83.4 ± 13.9 mmHg) after administration of OM/AML/HCTZ by 6.6 ± 13.9 mmHg, 7.0 ± 13.9 mmHg, 7.2 ± 13.8 mmHg, and 7.4 ± 13.6 mmHg at month 3, 6, 9, and 12, respectively (all *P*-value < 0.0001) (Fig. 2). The mean SBP/DBP was reduced to 132.1/76.8 mmHg at month 3; 130.6/75.8 mmHg at month 6; 130.0/75.3 mmHg at month 9; and 129.1/75.0 mmHg at month 12, respectively (Fig. 3).

**Fig. 4:**
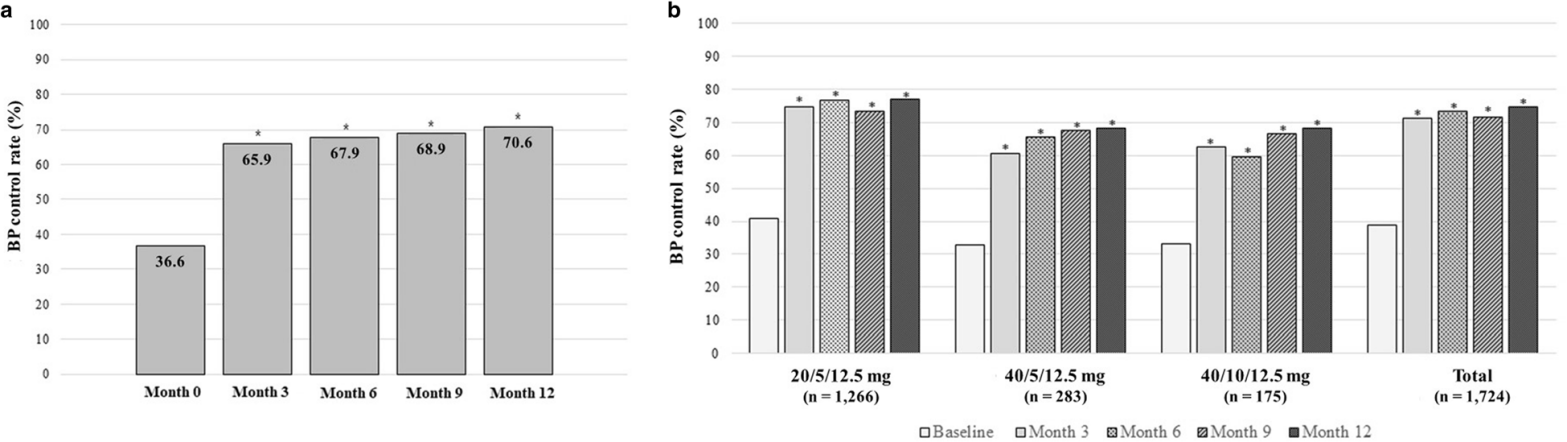
Blood pressure (BP) control rates. **a** BP control rates at month 3, 6, 9, and 12 after olmesartan/amlodipine/hydrochlorothiazide (OM/AML/HCTZ) administration in overall patients. **b** BP control rates at month 3, 6, 9, and 12 in patients treated only with OM/AML/HCTZ. Target BP, SBP/DBP < 140/90 mmHg). Effectiveness set (*n* = 3052). BP, blood pressure; SBP, systolic BP; DBP, diastolic BP. *P < 0.0001 (McNemar’s test)

The BP control rate of < 140/90 mmHg at month 3, 6, 9, and 12 after the administration of OM/AML/HCTZ was 65.9, 67.9, 68.9, and 70.6%, respectively (Fig. 4a). The mean MPR during the observation period was 0.96. NRS scores of patients’ and physician’s satisfaction with the use of SPC of OM/AML/HCTZ were 8.2 and 8.2 points at month 6; and 8.6 and 8.4 points at month 12, respectively.

### Subgroup analysis

In a subgroup analysis, the BP lowering effect of OM/AML/HCTZ was evaluated in 1724 patients who had maintained the baseline dose of OM/AML/HCTZ throughout the entire study period, without additional antihypertensive medications to control BP. The mean SBP was significantly reduced at all time points from baseline (142.1 ± 18.8 mmHg) by 13.3 ± 20.5 mmHg, 13.9 ± 20.3 mmHg, 13.3 ± 20.2 mmHg, and 13.6 ± 19.6 mmHg at month 3, 6, 9, and 12, respectively. The mean SBP in the subgroup of patients at each month was as follows: 128.8 ± 14.8 mmHg, 127.7 ± 14.3 mmHg, 127.8 ± 13.9 mmHg, and 127.4 ± 13.6 mmHg, respectively (all *P* < 0.0001) (Fig. S1). The mean baseline DBP was also significantly reduced at all time points by 6.9 ± 13.2 mmHg, 7.4 ± 13.5 mmHg, 7.2 ± 13.1 mmHg, and 7.5 ± 12.9 mmHg at month 3, 6, 9 and 12 from baseline (82.8 ± 13.7 mmHg). The mean DBP at each month was as follows: 75.8 ± 10.3 mmHg, 74.9 ± 10.1 mmHg, 74.7 ± 10.0 mmHg, and 74.5 ± 10.2 mmHg, respectively (all *P* < 0.0001) (Fig. S1). In addition, the overall BP control rate of this subgroup of patients (*n* = 1724) was 71.2, 73.1, 71.6, and 74.6% at month 3, 6, 9 and 12, respectively (Fig. 4b). The BP control rate achieved by each strength of OM/AML/HCTZ in the subgroup are as follows: 74.7, 76.6, 73.1, and 76.8% at month 3, 6, 9, and 12 in 20/5/12.5 mg group (*n* = 1266); 60.6, 65.7, 67.6, and 68.1% at month 3, 6, 9, and 12 in 40/5/12.5 mg group (*n* = 283); 62.5, 59.6, 66.4, and 68.2% at month 3, 6, 9, and 12 in 40/10/12.5 mg group, respectively (all *P* < 0.0001) (Fig. 4b).

Moreover, the change in the mean SBP/DBP relative to the baseline following OM/AML/HCTZ administration was examined in patients with DM (*n* = 983), CVD (*n* = 509), or RD (*n* = 128). The patients in the DM and CVD groups showed significant reductions in the mean SBP/DBP at month 3, 6, 9, and 12, relative to the baseline (all *P* < 0.0001). Moreover, the patients with RD showed significant reductions in SBP at all measured points during the study period with significant reductions in DBP observed at month 3 and 12, relative to the baseline SBP (all *P* < 0.0001) (Table S1).

The change in the mean SBP/DBP relative to the baseline after OM/AML/HCTZ administration was examined according to age groups (< 60 years, 60 ≤ & < 65 years, 65 ≤ & < 70 years, 70 ≤ & < 74 years, 75 ≤ & < 80 years, and > 80 years). Significant reductions in the mean SBP/DBP were observed at all measured points in all age groups (*P* < 0.0001 to 0.0003) (Table S2).

### Safety assessment

In the safety analysis set (*n* = 3370), AEs and serious AEs (SAEs) were reported by 26.8% (*n* = 902) and 4.8% (*n* = 163), respectively (Table 2). Dizziness was the most frequently reported AE (*n* = 183, 5.4%), followed by headache (*n* = 55, 1.6%), and arthralgia (*n* = 41, 1.2%). SAEs included angina pectoris (*n* = 10, 0.3%), pneumonia and chest pain (*n* = 8, 0.2%, for each SAE), and dizziness (*n* = 5, 0.2%).

**Table 2:** Summary of TEAE and ADR (safety set, *n* = 3370)

Variable	TEAE	ADR
Total	902 (26.8) [1727]	332 (9.9) [626]
Serious	163 (4.8) [225]	11 (0.3) [14]
Leading to drug withdrawal	255 (7.6) [307]	185 (5.5) [215]
Leading to death	5 (0.2) [7]	0 [0]

Data are presented as number (%) [case]

*TEAE* treatment-emergent adverse event, *ADR* adverse drug reaction

The discontinuation rate of OM/AML/HCTZ due to AEs in the safety analysis set was reported at 7.6% (*n* = 255) with dizziness being the most common (*n* = 87, 2.6%), followed by hypotension (*n* = 45, 1.3%) (Table 2). AEs that led to five deaths (0.2%) were not considered to be related to OM/AML/HCTZ treatment. All deaths resulted from non-cardiovascular causes including gastric cancer, malignant lung neoplasm, small-intestine carcinoma, pneumonia, septic shock, cerebrovascular accident and renal failure (*n* = 1, for each death).

ADRs with causal relationship to OM/AML/HCTZ were reported by 9.9% (*n* = 332). The most common ADR was dizziness (*n* = 106, 3.2%), followed by hypotension (*n* = 56, 1.7%). Serious ADRs (SADRs) were reported by 0.3% (*n* = 11) (Table 2). Of the 14 cases of SADRs, ten cases of SADRs that were categorized as ‘unclassified’ have remained at OM/AML/HCT treatment and were later reported as ‘recovered’. Four cases of SADRs including chest pain, hypotension, dizziness, and hyponatremia led to discontinuation of OM/AML/HCTZ treatment and were later reported as ‘recovered.’

The discontinuation rate of OM/AML/HCTZ due to ADRs was reported by 5.5% (*n* = 185). The most frequent ADRs causing withdrawal from the treatment were dizziness (*n* = 75, 2.2%), followed by hypotension (*n* = 45, 1.3%), and headache (*n* = 9, 0.3%). No ADR-related deaths occurred (Table 2).

## Discussion

This study prospectively evaluated the effectiveness and safety of the triple SPC of OM/AML/HCTZ in Korean hypertensive patients in a real clinical practice setting.

Following administration of OM/AML/HCTZ, significant reductions from baseline in the mean SBP/DBP were observed at all measured time points (month 3, 6, 9, and 12). The greatest reductions in both SBP and DBP were observed at month 3 and the reduced mean SBP/DBP was sustained close to < 130/80 mmHg throughout the 12-month observation period. These results were in accordance with the recommendations of current ESC/ESH hypertension guideline, where the BP goal of at least 130/80 mmHg is recommended in most patients, once SBP/DBP was safely controlled under the threshold of 140/90 mmHg [5]. The significant BP reductions were consistently observed across all age groups and risk factors (Tables S1, S2).

It is known that early and fast BP management is associated with more effective and lasting BP control and hence greater long-term clinical benefits. Previous studies have shown that achievement of BP control within 6 months is associated with significantly reduced incidence of cardiovascular outcomes [13]. In this study, OM/AML/HCTZ demonstrated a fast onset and long-lasting effect on BP control by achieving the control rate of 65.9% at month 3, which gradually increased to 70.6% until month 12 (Fig. 4a). Higher BP control rates were observed in the subgroup of patients (71.2 to 74.6%), further supporting the effectiveness of OM/AML/HCTZ for controlling BP without dose escalation or addition of other agents (Fig. 4b). These results were comparable to that reported in 12-week TRINITY study (70.0%) and in 40-week TRINITY-extension study (44.5 to 79.8%, depending on treatment dose) [8, 9]. According to 2018 KSH guidelines, 71% of patients treated with antihypertensive therapy achieved BP control and this was supported by the present real-world study [6].

Patient satisfaction to treatment is highly correlated with patient adherence and treatment success. In this study, patients indicated high levels of satisfaction with and adherence to OM/AML/HCTZ treatment (NRS of 8.2 to 8.6 and MPR of 0.96, respectively) which were possibly attributable to effective BP management throughout the treatment course.

Most hypertensive patients require more than single antihypertensive agent to control BP and often require concurrent use of three agents from different classes [3, 4]. In Korea, it appears that approximately 60% of patients take two or more antihypertensive medications and 17.7% of them are receiving three or more different classes [2]. Several SPCs have been released to improve medication adherence by reducing pill burden and complexity of dosing regimens, and have shown to increase the BP control rate [1, 14, 15]. OM/AML/HCTZ was the first triple SPC launched in Korea and the efficacy of the triple SPC of OM/AML/HCTZ has been well-demonstrated in previous RCTs [8–10].

RCTs have limitations with respect to representing a wide diversity of patients in the real clinical practice, although it is regarded as the most reliable research design owing to its strict patient selection criteria and blinding process. In real-world, the hypertension status varies according to race/ethnicity, however, only limited data on the Korean population have been available. In addition, since most RCTs are conducted over a short period of up to 3 to 6 months, the long-term effect of a treatment often remains to be determined. This study was conducted prospectively in a routine clinical setting over a period of 12 months, involving a wide variety of patient groups. Thus, it was less prone to have recall bias compared with retrospective studies.

The subgroup analysis of this real-world study revealed that the majority of patients (74.4%) had administered OM/AML/HCTZ at the standard dose of 20/5/12.5 mg. These patients were maintained on the same dose until month 12 and achieved the BP control rates greater than that reported in the previous global study where the patients were treated with the high dose of OM/AML/HCTZ 40/10/25 mg (73.1 to 76.8% vs. 69.9%, respectively) [8]. Furthermore, according to the study, which examined the long-term efficacy of OM/AML/HCTZ, the highest BP control rate at week 52 was achieved in the patients receiving 40/5/12.5 mg (79.8%) [9]. Taken together, for Korean patients in real-world practice, the standard dose of OM/AML/HCTZ (20/5/12.5 mg) was as effective option as the high dose OM/AML/HCTZ.

The present study was initially designed according to the 2014 Joint National Committee volume 8. During the study process, 2018 KSH and 2018 ESC/ESH guidelines have been updated, with greater emphasis on the use of SPC in hypertensive patients. In order to provide the RWE on the significance of the triple SPC strategy in Korean patients in accordance with the current hypertension guidelines, the analysis of the results of the present study were based on the 2018 KSH and 2018 ESC/ESH guidelines. This involved adjustment of age and target BP of the elderly group.

In this study, the most common AEs and ADRs included dizziness, headache, hypotension and arthralgia. A similar AE profile was observed in TRINITY study where dizziness (9.9%), peripheral edema (7.7%), and headache (6.4%) occurred most frequently [10]. No additional safety issue was identified that requires reassessment of the safety of OM/AML/HCTZ.

This study has the inherent limitations associated with multicenter, non-comparative, observational study design. The presence of missing data was unavoidable due to large datasets. However, the current study has strengths in its prospective study design and in that a large number of more than 3000 hypertensive patients in Korea were evaluated in a real clinical setting over a longer period than previous studies [11]. Therefore, this study contributed to establishing a rationale for use of the triple SPC in Korean patients and has proven that the standard dose of OM/AML/HCTZ is a safe and effective option for the majority of Korean patients with essential hypertension.

Considering previous studies of OM/AML/HCTZ that showed reduced cardiovascular events and mortality by early and long-term BP control, future research may investigate the long-term effectiveness of OM/AML/HCTZ on cardiovascular outcomes in Korean patients [16–18].

## Conclusions

The triple SPC of OM/AML/HCTZ significantly reduced the mean SBP/DBP relative to the baseline in Korean patients with essential hypertension. The BP lowering effects were maintained throughout the 12-month observation period along with high patient adherence and BP control rates. OM/AML/HCTZ was safe and well-tolerated in Korean patients.

## Supplementary Information

**Additional file 1: Supplementary Figure 1.** Time course change in the mean SPB/DBP at month 3, 6, 9 and 12 in patients treated only with OM/AML/HCTZ**. Supplementary Table 1.** Changes from baseline in the mean SBP/DBP at month 3, 6, 9 and 12 in patients with risk factors. **Supplementary Table 2.** Changes from baseline in the mean SBP/DBP at month 3, 6, 9 and 12 in patients with different age groups. **Supplementary Table 3**. Participating institutions.

## Data Availability

The datasets are not publicly available but are available from the corresponding author upon reasonable request.

## Abbreviations

ADRs: Adverse reactions
AEs: Adverse events
AML: Amlodipine
BP: Blood pressure
CVD: Cardiovascular disease
DBP: Diastolic blood pressure
DM: Diabetes mellitus
ESC: European Society of Cardiology
ESH: European Society of Hypertension
HCTZ: Hydrochlorothiazide
KSH: Korean Society of Hypertension
MPR: Medication possession ratio
NRS: Numeric rating scale
OM: Olmesartan
RCTs: Randomized-controlled trials
RD: Renal disease
RWE: Real-world evidence
SBP: Systolic blood pressure
SADRs: Serious adverse reactions
SAEs: Serious adverse events
SPC: Single-pill combination
