# Supplementary material for: Real-world evidence on the strategy of olmesartan-based triple single-pill combination in Korean hypertensive patients: a prospective, multicenter, observational study (RESOLVE-PRO)
Source: Clin Hypertens. 2021 Nov 1;27:21. doi: 10.1186/s40885-021-00177-z (PMC8559412; doi:10.1186/s40885-021-00177-z)
Supplement: Supplementary file 1 — Additional file 1: Supplementary Figure 1. Time course change in the mean SPB/DBP at month 3, 6, 9 and 12 in patients treated only with OM/AML/HCTZ. Supplementary Table 1. Changes from baseline in the mean SBP/DBP at month 3, 6, 9 and 12 in patients with risk factors. Supplementary Table 2. Changes from baseline in the mean SBP/DBP at month 3, 6, 9 and 12 in patients with different age groups. Supplementary Table 3. Participating institutions. [file 40885_2021_177_MOESM1_ESM.docx]

|  |  | **DM**  **(n = 983)** | **Non-DM**  **(n = 2,069)** | **RD**  **(n = 128)** | **Non-RD**  **(n = 2,924)** | **CVD**  **(n = 509)** | **Non-CVD**  **(n = 2,543)** |
| --- | --- | --- | --- | --- | --- | --- | --- |
|  | SBP [mean (SD)] | 141.5 (18.2) | 144.1 (19.8) | 142.3 (17.5) | 143.3 (19.4) | 140.1 (18.2) | 143.9 (19.5) |
| Baseline |  |  |  |  |  |  |  |
|  | DBP [mean (SD)] | 80.6 (12.7) | 84.7 (14.3) | 79.3 (14.2) | 83.6 (13.9) | 80.3 (12.1) | 84.0 (14.2) |
| 3 Month | SBP [mean (SD)] | 133.6 (16.4) | 131.5 (16.8) | 134.8 (15.3) | 132.0 (16.8) | 130.8 (16.1) | 132.4 (16.8) |
|  | CFB | -8.4 (18.6)* | -12.9 (21.0)* | -8.2 (17.1)¶ | -11.6 (20.5)* | -9.7 (19.2)* | -11.8 (20.6)* |
|  |  |  |  |  |  |  |  |
|  | DBP [mean (SD)] | 76.3 (11.2) | 77.0 (11.0) | 75.9 (11.4) | 76.8 (11.1) | 75.1 (10.0) | 77.1 (11.3) |
|  | CFB | -4.4 (13.1)* | -7.6 (14.1)* | -2.6 (11.0)¶ | -6.7 (14.0) | -5.1 (12.6)* | -6.9 (14.1)* |
| 6 Month | SBP [mean (SD)] | 131.4 (16.3) | 130.2 (16.0) | 132.4 (17.8) | 130.5 (16.0) | 129.8 (15.3) | 130.7 (16.3) |
|  | CFB | -9.7 (19.3)* | -13.6 (21.3)* | -7.8 (19.4)¶ | -12.5 (20.8)* | -9.7 (19.4)* | -12.9 (21.0)* |
|  |  |  |  |  |  |  |  |
|  | DBP [mean (SD)] | 74.9 (10.9) | 76.3 (11.1) | 75.9 (13.0) | 75.8 (11.0) | 74.4 (9.5) | 76.2 (11.3) |
|  | CFB | -5.5 (13.5)* | -7.8 (14.1)* | -1.6 (13.1)* | -7.3 (13.9)* | -5.4 (12.0)* | -7.4 (14.3)* |
| 9 Month | SBP [mean (SD)] | 131.2 (15.1) | 129.5 (15.5) | 130.6 (16.9) | 130.0 (15.3) | 127.2 (14.4) | 130.7 (15.6) |
|  | CFB | -9.7 (19.3)* | -13.6 (21.2)¶ | -9.0 (18.2)¶ | -12.5 (20.8)* | -11.6 (18.6)* | -12.5 (21.1)* |
|  |  |  |  |  |  |  |  |
|  | DBP [mean (SD)] | 75.3 (10.5) | 75.4 (10.5) | 74.7 (11.9) | 75.4 (10.5) | 73.3 (9.4) | 75.8 (10.7) |
|  | CFB | -5.0 (13.3)* | -8.3 (13.9)* | -2.9 (14.0)¶ | -7.4 (13.8)* | -6.6 (12.1)¶ | -7.4 (14.2)* |
| 12 Month | SBP [mean (SD)] | 129.9 (15.5) | 128.7 (14.2) | 130.0 (15.0) | 129.0 (14.6) | 127.4 (14.9) | 129.5 (14.6) |
|  | CFB | -10.3 (19.2)* | -14.1 (20.8)¶ | -9.9 (17.0)¶ | -13.0 (20.5)* | -11.3 (19.6)* | -13.2 (20.5)* |
|  |  |  |  |  |  |  |  |
|  | DBP [mean (SD)] | 73.5 (10.6) | 75.8 (10.7) | 73.5 (11.1) | 75.1 (10.7) | 73.6 (10.0) | 75.3 (10.9) |
|  | CFB | -6.7 (13.6)* | -7.8 (13.5)* | -4.0 (13.9)¶ | -7.6 (13.5)* | -6.0 (12.7)* | -7.7 (13.7)* |

Effectiveness analysis set (n = 3,052)

SBP: systolic blood pressure, DBP: diastolic blood pressure, W: Wilcoxon signed rank test, CFB: change from baseline, DM: diabetes mellitus, RD: renal disease, CVD: cardiovascular diseases

***** P-value <0.0001 (w), ¶ P-value <0.0001(t)

| **Age < 60**  **(n = 1,123)** | | | **60 ≤ Age < 65**  **(n = 379)** | **65 ≤ Age < 70**  **(n = 458)** | **70 ≤ Age < 75**  **(n = 468)** | **75 ≤ Age < 80**  **(n = 420)** | **> 80 Age**  **(n = 204)** |
| --- | --- | --- | --- | --- | --- | --- | --- |
| n = 1,123 | | | n = 379 | n = 458 | n = 468 | n = 420 | n = 204 |
| 145.2 (19.9)/ | | | 140.1 (18.2)/ | 142.4 (18.5)/ | 141.9 (18.1)/ | 143.2 (19.7)/ | 144.6 (21.5)/ |
|  | [mean (SD)] | 89.2 (14.4) | 82.8 (12.6) | 81.0 (12.4) | 78.8 (11.8) | 78.8 (12.4) | 77.6 (13.3) |
| 3 Month |  | n = 1,087 | n = 368 | n = 428 | n = 442 | n = 403 | n = 201 |
|  | SBP/DBP | 133.6 (17.2)/ | 130.4 (16.9)/ | 130.5 (14.3)/ | 131.9 (16.5)/ | 130.2 (16.1)/ | 135.1 (18.8)/ |
|  | [mean (SD)] | 80.9 (11.5) | 76.1 (9.9) | 75.1 (9.8) | 73.9 (10.0) | 73.0 (10.2) | 73.5 (10.4) |
|  |  | n = 999 | n = 337 | n = 395 | n = 412 | n = 373 | n = 181 |
|  | CFB of SBP/DBP | -12.3 (21.0)*/ | -10.3 (19.7)*/ | -11.9 (18.9)*/ | -10.0 (20.2)*/ | -12.4 (20.1)*/ | -9.5 (21.7)*/ |
|  |  | -8.2 (14.9)* | -6.9 (13.7)* | -5.9 (12.4)* | -5.0 (12.8)* | -5.7 (13.7)* | -3.9 (13.5)** |
| 6 Month |  | n = 886 | n = 318 | n = 384 | n = 400 | n = 342 | n = 155 |
|  | SBP/DBP | 130.9 (16.2)/ | 129.6 (15.4)/ | 129.9 (16.3)/ | 130.9 (16.1)/ | 129.7 (15.8)/ | 132.9 (16.8)/ |
|  | [mean (SD)] | 79.9 (11.6) | 75.8 (10.5) | 74.3 (10.1) | 72.9 (9.6) | 72.3 (9.5) | 71.6 (10.1) |
|  |  | n = 845 | n = 304 | n = 361 | n = 379 | n = 329 | n = 146 |
|  | CFB of SBP/DBP | -13.5 (21.2)*/ | -10.3 (19.8)*/ | -12.4 (20.3)*/ | -10.6 (18.9)*/ | -13.4 (21.2)*/ | -12.0 (24.1)*/ |
|  |  | -8.5 (15.3)* | -6.3 (13.7)¶ | -6.6 (13.4)* | -5.6 (11.9)* | -6.3 (12.7)* | -6.3 (14.3)* |
| 9 Month |  | n = 765 | n = 285 | n = 323 | n = 354 | n = 291 | n = 129 |
|  | SBP/DBP | 130.7 (15.6)/ | 128.3 (14.7)/ | 130.1 (14.7)/ | 129.9 (15.1)/ | 129.3 (15.6)/ | 131.8 (17.7)/ |
|  | [mean (SD)] | 79.4 (10.3) | 75.6 (9.7) | 74.3 (9.7) | 72.1 (10.1) | 71.4 (9.7) | 71.5 (10.3) |
|  |  | n = 723 | n = 270 | n = 311 | n = 341 | n = 280 | n = 120 |
|  | CFB of SBP/DBP | -13.1 (21.3)*/ | -11.3 (20.2)¶/ | -12.2 (20.0)*/ | -10.9 (19.4)*/ | -13.6 (21.4)*/ | -11.2 (21.2)*/ |
|  |  | -8.5 (14.8)* | -6.7 (13.4)¶ | -6.5 (13.1)* | -6.4 (12.4)* | -7.1 (13.5)* | -5.2 (14.2)↑ |
| 12 Month |  | n = 784 | n = 283 | n = 335 | n = 364 | n = 308 | n = 124 |
|  | SBP[mean (SD)] | 123.0 (14.3)/ | 128.0 (14.6)/ | 128.0 (14.3)/ | 127.4 (14.7)/ | 130.2 (15.2)/ | 130.9 (15.7)/ |
|  |  | 79.3 (10.4) | 76.1 (9.2) | 73.73 (10.0) | 71.2 (9.8) | 71.0 (10.6) | 70.4 (10.9) |
|  |  | n = 763 | n = 275 | n = 331 | n = 55 | n = 298 | n = 122 |
|  | CFB of SBP/DBP | -13.6 (20.7)*/ | -11.3 (20.1)¶/ | -13.2 (19.2)*/ | -13.1 (19.9)*/ | -12.7 (21.1)*/ | -10.1 (21.8)¶/ |
|  |  | -8.4 (14.6)* | -6.1 (13.1)* | -6.6 (12.3)* | -7.2 (12.4)* | -7.8 (13.4)* | -5.9 (14.4)* |

Baseline SBP/DBP

Effectiveness analysis set (n=3,052), SBP: systolic blood pressure, W: Wilcoxon signed rank test, t; paired t-test, CFB: change from baseline

* P-value <0.0001(w) ** P-value 0.0003 (w) ↑P-value 0.0002(w) ¶ P-value <0.0001(t)


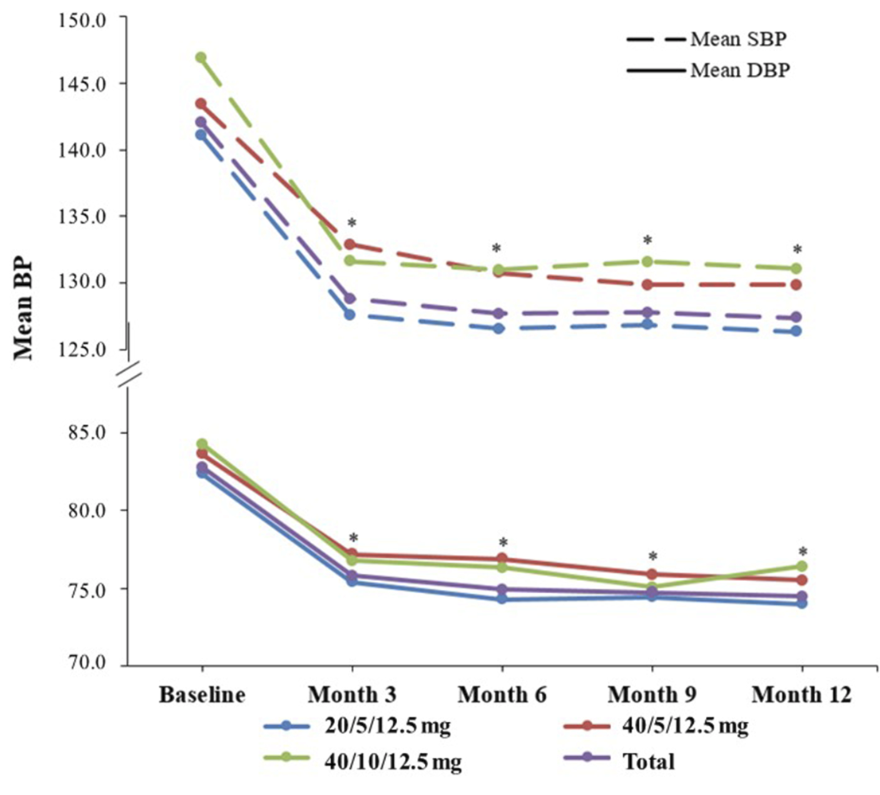


Subgroup analysis set (n = 1,724) from the effectiveness set (n = 3,052).

BP: blood pressure, SBP: systolic blood pressure, DBP: diastolic blood pressure.

*P<0.0001 (Wilcoxon signed rank test)

| No. | Investigator | Institution | | IRB No. |
| --- | --- | --- | --- | --- |
| 1 | Chong-Jin Kim | Kyung Hee University Hospital at Gangdong | General | 2016-08-009 |
| 2 | Sang-Hyun Ihm | Bucheon St. Mary’s Hospital | General | HC16OSME0031 |
| 3 | Gee Hee Kim | St. Vincent’s Hospital | General | VC16OSME0098 |
| 4 | Sang Min Park | Hallym University Chuncheon Sacred Heart Hospital | General | 2016-47 |
| 5 | Bum-Kee Hong | Gangnam Severance Hospital | Tertiary | 3-2016-0312 |
| 6 | Chang Hoon Lee | Veterans Health Service Medical Center | General | 2016-05-017 |
| 7 | Sang Hyun Lee | Pusan National University Yangsan Hospital | Tertiary | 02-2016-022 |
| 8 | Dae-Il Chang | Kyung Hee University Hospital | Tertiary | 2016-05-102 |
| 9 | Sung-Pil Joo | Chonnam National University Hospital | Tertiary | CNUH-2016-100 |
| 10 | Sang-Chan Lee | Dong-Eui Hospital | General | DEMC-2017-01 |
| 11 | Yong-Ho Lee | Severance Hospital | Tertiary | 4-2016-0464 |
| 12 | Dong Woon Jeon | National Health Insurance Service Ilsan Hospital | General | 2016-04-016 |
| 13 | Kyung Tae Jung | Eulji University Hospital | General | 2016-04-008 |
| 14 | Yun Sung Kim | Chosun University Hospital | Tertiary | 2016-04-013 |
| 15 | Seong-Ill Woo | Inha University Hospital | Tertiary | 2016-07-0004 |
| 16 | Kyounghoon Lee | Gacheon University Gil Medical Center | Tertiary | GBIRB2016-218 |
| 17 | Jung Ho Heo | Kosin University Gospel Hospital | General | 2016-04-020 |
| 18 | Sang-Ho Park | Soonchunhyang University Cheonan Hospital | Tertiary | 2016-04-033 |
| 19 | Euy Jin Choi | Bucheon St. Mary’s Hospital | General | HC16MSME0084 |
| 20 | Sun Ho Hwang | Gwangju Veterans Hospital | General | 2016-04-01 |
| 21 | Yonh-Gu Chung | Korea University Anam Hospital | Tertiary | AN-16099-001 |
| 22 | Young Jin Youn | Wonju Severance Christian Hospital | Tertiary | CR316021 |
| 23 | Eul-Soon Im | Dongsuwon General Hospital | General | DSW-16-002 |
| 24 | Jong Sam Baik | Inje University Sanggye Paik Hospital | General | SGPAIK 2016-05-020 |
| 25 | Nack-Cheon Choi | Gyeongsang National University Hospital | Tertiary | 2016-12-006 |
| 26 | Jin Bae Lee | Daegu Catholic University Medical Center | Tertiary | CR-16-068-L |
| 27 | Kyu-Hyung Ryu | Hallym University Dongtan Sacred Heart Hospital | General | 2016-157-S |
| 28 | Ji-Yong Jang | Konkuk University Chungju Hospital | General | KUCH 2016-04-017 |
| 29 | Sung-Ji Park | Samsung Medical Center | Tertiary | 2016-04-154 |
| 30 | Dong-Ick Shin | Chungbuk National University Hospital | Tertiary | 2016-04-011 |
| 31 | Jin Oh Na | Korea University Guro Hospital | Tertiary | KUGH16168-001 |
| 32 | Yun Jae Chung | Chung-Ang University Hospital | Tertiary | C2016110(1853) |
| 33 | Su Hyun Kim | Carollo Hospital | General | SCH2016-090 |
| 34 | Jong-Young Lee | Kangbuk Samsung Hospital | Tertiary | 2016-04-032 |
| 35 | Yoon-Sang Oh | Yeouido St. Mary’s Hospital | General | SC16OSME0063 |
| 36 | Jong Hwan Choi | Daegu Fatima Hospital | General | DFH16MRSO302 |
| 37 | Hye Sun Seo | Soonchunhyang University Bucheon Hospital | Tertiary | 2016-04-005 |
| 38 | Su Kyoung Kwon | Kosin University Gospel Hospital | General | 2016-12-014 |
| 39 | Hyung-Jun Kim | Dongkang Medical Center | General | 2016-08-02 |
| 40 | Jin-Sun Park | Ajou University Hospital | Tertiary | MED-OBS-16-487 |
| 41 | Kyoung-Ha Park | Hallym University Sacred Heart Hospital | Tertiary | 2016-S035 |
| 42 | Kyoung-Soo Lee | Samsung Changwon Hospital | Tertiary | 2016-SCMC-028-00 |
| 43 | Sung Chang Chung | Dongkang Medical Center | General | 2016-06-01 |
| 44 | Han-Jin Cho | Pusan National University Hospital | Tertiary | D-D-1607-011-054 |
| 45 | Chang-Min Lee | Dankook University Hospital | Tertiary | 2016-04-022 |
| 46 | Sung-Rae Kim | Bucheon St. Mary’s Hospital | General | HC16MSME0080 |
| 47 | Jinkwon Kim | CHA Bundang Medical Center | General | 2016-04-008 |
| 48 | Ik Seong Park | Bucheon St. Mary’s Hospital | General | HC16MSME0033 |
| 49 | Pil-Wook Chung | Kangbuk Samsung Hospital | Tertiary | 2016-04-033 |
| 50 | Sung-Rae Cho | Changwon Fatima Hospital | General | 2016-03-02 |
| 51 | Hyun-Seung Kang | Seoul National University Hospital | Tertiary | H-1609-099-793 |
| 52 | Seung Jin Lee | Soonchunhyang University Cheonan Hospital | Tertiary | 2016-12-012 |
| 53 | Kyungil Park | Dong-A University Hospital | Tertiary | DAUHIRB-TEMP-16-170 |
| 54 | Si Won Lee | Busan Veterans Hospital | General | 2016-06 |
| 55 | Jang-Won Son | Yeungnam University Medical Center | Tertiary | 2016-04-009 |
| 56 | Soo Kyoung Kim | Gyeongsang National University Hospital | Tertiary | 2016-04-006 |
| 57 | Joong-Hwa Chung | Chosun University Hospital | Tertiary | 2016-04-016 |
| 58 | Jin-Soo Byon | Soonchunhyang University Gumi Hospital | General | 2016 · 15 |
| 59 | Hong Euy Lim | Korea University Guro Hospital | Tertiary | 2016-12-0011 |
| 60 | Bum-Tae Kim | Soonchunhyang University Bucheon Hospital | Tertiary | 2016-07-006 |
| 61 | Yu Jeong Choi | Eulji University Hospital | General | 2016-04-009 |
| 62 | Soe Hee Ann | Ulsan University Hospital | Tertiary | 2016-04-004 |
| 63 | Sang Min Sung | Pusan National University Hospital | Tertiary | D-1604-015-051 |
| 64 | Jun-Hee Lee | Kangdong Sacred Heart Hospital | General | 2016-07-013 |
| 65 | Deuk-Young Nah | Dongguk University Gyeongju Hospital | General | 110757-201605-HR-03-01 |
| 66 | Jung-Han Kim | Sung Ae General Hospital | General | SA2016-08 |
